# Furin as a Novel Pan-Viral Therapeutic Target: Implications for Dengue and SARS-CoV-2

**DOI:** 10.3390/v18050509

**Published:** 2026-04-29

**Authors:** Lina Shalaby, Yaman Al-Haneedi, Alaa Abdelhamid, Hadi Yassine, Mohamed M. Emara

**Affiliations:** 1 School of Medicine, Wayne State University, Detroit, MI 48201, USA; lshalaby@wayne.edu; 2 Basic Medical Sciences Department, College of Medicine, Qatar University Health, Qatar University, Doha P.O. Box 2713, Qatar; ya2007037@qu.edu.qa (Y.A.-H.); aa2106093@student.qu.edu.qa (A.A.); 3 Biomedical Research Center, Qatar University Health, Qatar University, Doha P.O. Box 2713, Qatar; hyassine@qu.edu.qa; 4 College of Health Sciences, Qatar University, Doha P.O. Box 2713, Qatar

**Keywords:** dengue virus, SARS-CoV-2, co-infection, vaccines, direct-acting antivirals, host-directed antivirals, protease inhibitors, furin

## Abstract

Dengue virus (DENV) and SARS-CoV-2 are emerging viral pathogens that share overlapping clinical features, including fever, fatigue, and respiratory symptoms, complicating differential diagnosis in endemic regions. Their co-circulation has increased the risk of co-infections, which may result in unpredictable disease progression, increased morbidity, and mortality. This overlap presents a significant challenge in managing outbreaks, as both viruses pose a major public health threat. Vaccines and direct-acting antivirals may be rendered ineffective by viral mutations, making it difficult to address evolving strains. Host-directed antivirals offer a promising alternative, potentially maintaining efficacy against a multitude of variants. Both DENV and SARS-CoV-2 rely on host proteases for viral maturation and entry, with furin playing a crucial role in viral glycoprotein cleavage. In DENV, furin cleaves the prM protein, facilitating virion maturation, while in SARS-CoV-2, the polybasic furin cleavage site in the spike protein enhances viral entry. This makes furin a compelling pan-viral target, where inhibiting furin could reduce viral fitness without relying on viral mutations. This review highlights the therapeutic rationale for targeting furin and discusses luteolin, a furin inhibitor showing antiviral activity against both viruses. Furin-targeted therapies may offer a durable antiviral strategy effective across DENV serotypes, SARS-CoV-2 variants, and co-infection settings.

## 1. Introduction

*Orthoflavivirus denguei* (DENV) is an arthropod-borne viral disease caused by infection with any of the four-dengue virus serotypes (DENV-1 to DENV-4) [1]. DENVs are enveloped single-stranded positive-sense RNA viruses that are spherical in shape and belong to the *flaviviridae* family [2,3]. They are primarily transmitted through the *Aedes aegypti* and *Aedes albopictus* mosquitos, making them a common cause of mosquito-borne viral disease in tropical and subtropical regions [1]. In 2024, the incidence of dengue was reported to be 14.6 million by the World Health Organization (WHO), highlighting a 30-fold increase from the 505,430 cases recorded in 2000 [4]. Rising temperature and prolonged mosquito breeding seasons, due to global warming, enable the expansion of mosquito habitats into previously temperate regions [5]. DENV infections result in a wide range of symptoms, ranging from a flu-like syndrome, termed dengue fever (DF), to the potentially fatal dengue shock syndrome (DSS) [6]. Symptoms of DF include fever, nausea, vomiting, rash, and myalgia, while DSS results in severe bleeding and shock [6]. Such severe manifestations are associated with mortality, ranging from 5% in those who receive active treatment to 20% in patients who do not [7]. Moreover, it is important to note that immunity to a singular DENV serotype does not confer cross-protection against other serotypes [8]. Therefore, severe DF may manifest in individuals experiencing secondary infections with heterotypic DENV strains, as well as in infants born to dengue-immune mothers with primary anti-DENV antibody responses [6]. Consequently, the greatest DENV burden falls on pediatric, adolescent, and young adult populations [9]. The rising geographic spread of DENV, coupled with the lack of effective treatment options, highlights an urgent need for antiviral therapies, a demand that is expected to increase significantly in the coming years.

**Figure 1:**
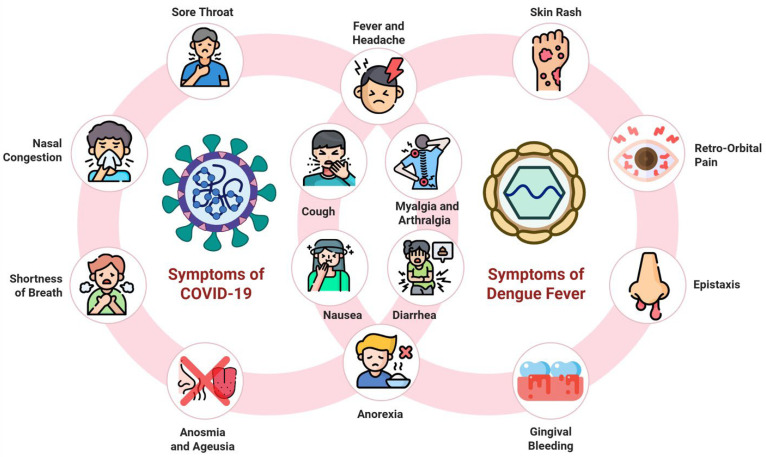
Overlapping clinical manifestations of COVID-19 and DF. Both diseases share several nonspecific symptoms, including fever, myalgia, headache and cough. This makes early clinical differentiation challenging, especially in regions where both viruses co-circulate, thereby complicating diagnosis and delaying appropriate management. Created in BioRender. Abdelhamid, A. (2026) https://BioRender.com/mg71rkj (accessed on 1 April 2026).

DF may be complicated by coinfection with severe acute respiratory syndrome cornavirus-2 (SARS-CoV-2), an enveloped, spherical, single-stranded positive-sense RNA virus belonging to the subgenus Sarbecovirus within the genus Betacoronavirus of the family Coronaviridae [10]. It primarily transmitted through droplets and aerosols [11,12,13,14]. Infection with SARS-CoV-2 manifests in COVID-19 a syndrome characterized by nausea, anorexia, fever, headache, and cough amongst other features. COVID-19, which remains a global concern, as ongoing genetic mutations enable it to evade established immune responses, thereby sustaining its continued circulation. According to the WHO, there has been an increase in the activity levels of SARS-CoV-2 with test positivity reaching a staggering 11% since the start of 2025 [15]. Such levels have not been reported since July of 2024, emphasizing that SARS-CoV-2 remains to be a threat to global health and a contributor to morbidity and mortality [15]. The persistent threat posed by SARS-CoV-2 may be partly due to the constant emergence of new variants driven by rapid mutation rates, leading to altered spike proteins capable evading immune responses generated by prior infection or immunization [16]. Indeed, variants such as Alpha, Beta, Gamma, Delta and Omicron, have all been shown to compromise the effectiveness of newer vaccine strategies thereby contributing ongoing global transmission [17]. The resurgence of variant-driven outbreaks increases risk of coinfection with other viral disease such as DENV. Coinfection represents significant clinical challenge overcome, as COVID-19 and DF may appear strikingly similar with overlapping clinical features including anorexia, cough, fever and nausea, thereby complicating accurate differentiation (Figure 1) [13,18]. Co-infection with both viruses is becoming increasingly recognized. However, large-scale studies examining the global incidence and prevalence of such co-infections remain limited. This gap in the literature may partly reflect the relatively recent emergence of SARS-CoV-2 in 2019, as well as challenges in differential diagnosis due to overlapping clinical features. Nevertheless, several smaller studies have begun to explore this issue. For instance, a study conducted by Nadim et al. reported that 31% of patients (*n* = 2458) initially diagnosed with either DF or COVID-19 were subsequently identified as being co-infected with both DENV and SARS-CoV-2 [19]. The substantial size of this cohort underscores the clinical relevance of co-infection and highlights the urgent need for therapeutic strategies capable of targeting both viruses simultaneously

The rising global threat posed by DENV and SARS-CoV-2 maybe addressed with antiviral therapy. Antivirals are broadly classified into two classes: direct-acting antivirals (DAAs) and host-directed antivirals (HDAs) [20]. DAAs are viral specific and work via direct inhibition of viral proteins involved in viral replication and maturation. HDAs, on the other hand, work via inhibition of host proteins used by viruses during replication and maturation [20]. Several DAAs such as Remdesvir, Paxlovid and Lagevrio have been approved for the treatment of SARS-CoV-2 infection. Yet, no comparable therapeutic agents currently exist for DENV [21,22]. To date, the management of DENV infection remains largely supportive focusing only on symptom reduction and complication prevention [21]. The lack of effective antivirals reflects several obstacles inherent to the process of antiviral development. A major contributing factor is the rapid mutation rates characteristic of many RNA viruses, including SARS-CoV-2 and DENV [23,24]. By virtue of these rapid mutation rates, viruses are able to alter their antigenic properties rendering single-target antivirals, such as those targeted against viral polymerases or structural proteins, ineffective overtime [25]. While highly specific antivirals allow for potent targeting and inactivation of viral infection and replication, their specificity makes them vulnerable to resistance which may arise from any minor mutation. This obstacle is further compounded by the fact that numerous antiviral candidates with strong *in vitro* efficacy proved not applicable in clinical application, possibly due to their narrow therapeutic windows yielding unacceptable safety profiles. Furthermore, the development of virus-specific agents is hindered by significant regulatory, economic, and logistical barriers, including high costs, long development timelines, and the need for large, well-powered clinical trials, often within the unpredictable context of outbreak settings [26,27,28]. Collectively, these challenges highlight the need for new antivirals strategies that target host-cell factors, which are not subject to mutations driven by viral replication [29]. The added benefit of such approach is the fact that multiple viruses resort to the utilization of the exact same host factor [30,31]. Consequently, targeting host factors offers the potential to inhibit a wide range of viruses simultaneously using a single therapeutic agent, thereby paving the way for the development of broad-spectrum antivirals [30].

One such host-cell factor utilized by both SARS-CoV-2 and DENV is furin. Furin is an endoprotease localized within the trans-Golgi network that plays a crucial role in various physiological processes [32]. For example, it is involved in activation of several prohormones and proproteins into their biologically active forms including hormones, cytokines, and growth factors, including glucagon and transforming growth factor beta (TGF-β), all of which are essential for maintaining homeostasis [33]. Beyond its role in physiological processes, the proteolytic function of furin is exploited by several viruses, including SARS-CoV-2 and DENV [34]. In SARS-CoV-2, furin mediates cleavage of the spike (S) protein at the S1/S2 site, a step necessary for viral fusion and entry into host cells [35]. In DENV, furin cleaves the premembrane (prM) protein on immature viral particles, a process required for virion maturation [36]. Without this cleavage, immature viral particles incapable of host-cell infection are produced [36]. Because furin is critical to the maturation and infectivity of both SARS-CoV-2 and DENV, it represents a promising target for the development of broad-spectrum antiviral agents [31,37]. Targeting furin offers two major advantages. First, as a host-cell factor, it may be less subject to viral mutational escape, thereby reducing the chances of viral drug resistance [25,30]. Second, a single therapeutic agent directed against furin could theoretically inhibit multiple viruses that utilize it during their replication cycle [31]. This approach may be particularly beneficial in clinical contexts where COVID-19 and DF present with overlapping symptoms or in cases of co-infection, where a furin-targeted antiviral could provide effective coverage against both pathogens [34]. Consequently, targeting such common host factors has the potential to inhibit a wide range of viruses using a single broad-spectrum antiviral agent.

Several studies have highlighted the potential of direct furin inhibition as a therapeutic strategy for both DF and COVID-19, supported by preclinical studies investigating various furin inhibitors against DENV and SARS-CoV-2 infection individually. Despite this, there remains to be a limited number of studies exploring the role of furin inhibitors in cases of SARS-CoV-2 and DENV co-infection. Moreover, while existing literature predominantly emphasizes the benefits of furin inhibition, its potential harm has been insufficiently addressed. Therefore, this review aims to evaluate the therapeutic potential of furin inhibitors in SARS-CoV-2 and DENV coinfection, while also discussing the potential risks associated with furin blockade that may limit their use in clinical practice.

## 2. Furin: Structure, Function, and Expression

Furin is a calcium-dependent serine protease part of the proprotein convertase (PCs) family [38]. As of date, the PC family contains nine members, as follows: PC1/3, PC2, furin, PC4, PC5/6, PACE4, PC7, subtilisin kexin isozyme 1/site 1 protease (SKI-1/S1P), and PC subtilisin/kexin type 9 (PCSK9) [39]. However, relative to the other PC members, furin (PCSK3) is the first identified and most extensively characterized, making it an ideal candidate for antiviral treatment [38]. Furin is ubiquitously expressed across various tissues and localized in the *trans*-Golgi network/endosomal system that proteolytically activates several precursor protein substrates into mature proteins in eukaryotic cells [40]. In healthy cells, furin has various cellular functions, namely involving embryogenesis and homeostasis [40]. For instance, furin activates transforming growth factor (TGF-β), which is responsible for regulating cell differentiation, proliferation, and tissue development [41]. Moreover, furin plays an essential homeostatic role through prohormone processing, cleaving many substrates such as blood clotting factors, pro-β-nerve growth factor (pro-β-NGF), and the insulin pro-receptor, thus regulating hormonal signaling and metabolic balance [40,42]. Beyond its role in homeostatic functions, furin modulates tissue remodeling and facilitates cancer cell metastasis through its processing of matrix metalloproteinases (MMPs) [43]. MMPs are overexpressed by malignant cells and enzymatically degrade surrounding extracellular matrix allowing for movement through tissues and subsequent spillage into lymphatic/blood vessels [40,44]. Increased levels of furin results in increased levels of membrane type-1-matrix metalloproteinase (MT1-MMP), which is linked to increased aggressiveness of head and neck squamous cell carcinomas (HNSCC) and non-small-cell lung cancers (NSCLC) [45]. This connection suggests that in adults, the detrimental impact of high furin levels, particularly its role in promoting cancer progression, may outweigh its beneficial effects. Additionally, although furin knockout is lethal in the context of embryonic cells, its knockout does not result in lethality in differentiated somatic cells, remaining a promising antiviral therapeutic target for humans [46]. This resilience in somatic cells is key, as many dangerous viruses, from influenza to coronaviruses, hijack the host’s furin to activate themselves and spread. Consequently, inhibiting furin in an adult presents a promising strategy to develop broad-spectrum antiviral drugs, effectively disabling a wide range of viruses without causing harm to the patient.

## 3. Role of Furin in Viral Pathogenesis

Various enveloped viruses depend on furin or furin-like proprotein convertases to cleave the viral glycoprotein precursor [47,48]. Cleavage by furin typically occurs in the trans-Golgi network during biosynthesis or at the plasma membrane or endosomes to prime the fusion machinery so that subsequent triggers can fully expose the viral fusion peptide and drive membrane fusion and viral entry into cells [49]. For class-I fusion systems, such as *Alphainfluenzavirus Influenzae* (Influenza A) HA0, *Lentivirus humimdef1* (HIV-1) gp160, paramyxovirus F0 and coronavirus S glycoproteins, furin-mediated cleavage splits a single precursor into two subunits that remain associated with the new N-terminus of the membrane-anchored subunit, containing the fusion peptide [49]. Consequently, furin priming lowers the energy barrier for the large-scale conformational refolding that drives membrane merging [49].

During influenza A virus replication, the HA glycoprotein must be cleaved to become fusion-competent [50]. Due to the polybasic HA cleavage site (H5/H7), there is a shift from trypsin-like airway proteases to the usage of ubiquitously expressed furin-like convertases in infected cells, enabling systemic infection in poultry and increasing virulence [50]. Similarly, in HIV-1 replication, the env precursor protein is synthesized as a trimeric gp160 in the ER, glycosylated and traffics through the Golgi/TGN where a host proprotein convertase, predominantly furin, cleaves it into gp120 (surface subunit) and gp41 (transmembrane subunit). The subunits remain non-covalently associated on virions and infected cells. Blocking this cleavage by furin inhibitors results in uncleaved gp160 that bind CD4 T cells but are essentially non-infectious. Classic gain-/loss-of-function studies first established this furin-dependent activation, and multiple follow-ups have confirmed the functional role of furin for HIV glycoprotein cleavage [51].

## 4. Role of Furin in SARS-CoV-2 Infection

**Figure 2:**
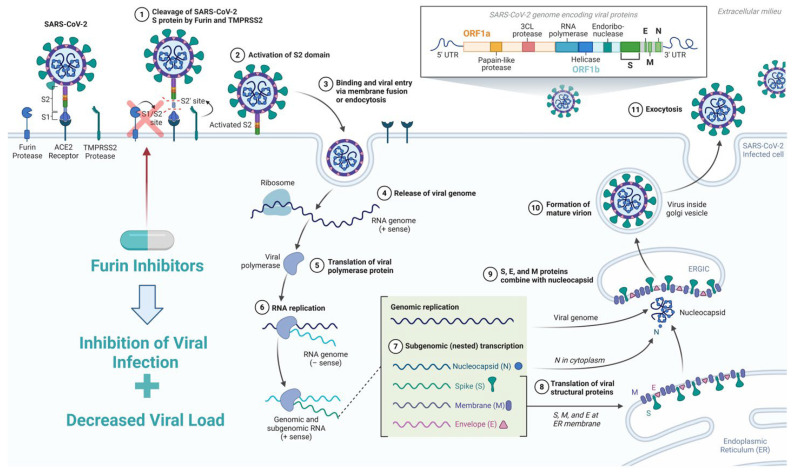
Role of furin protease in the SARS-CoV-2 viral life cycle. Furin plays a crucial role in the activation of the viral spike (S) glycoprotein through cleavage at the S1/S2 junction site. This activation is essential in the replication cycle of SARS-CoV-2, enhancing viral infectivity and cell–cell fusion, contributing to efficient viral entry and spread. Furin inhibition reduces SARS-CoV-2 infectivity, highlighting furin as a potential therapeutic target in antiviral drug development. Created in BioRender. Abdelhamid, A. (2026) https://BioRender.com/ojbp5ek (accessed on 1 April 2026).

Furin plays a crucial role in the SARS-CoV-2 infection and viral entry into alveolar cells [52,53]. At the surface of the viral envelope, furin recognizes the S1/S2 cleavage site of the S-glycoprotein [54]. This cleavage exposes the binding and fusion domains, facilitating viral entry into the host cell, priming the spike protein for a second, entry-stage cut at S2′ by TMPRSS2 or endosomal cathepsins (Figure 2) [54,55]. Substantiating this, Papa et al. (2021) demonstrated that furin knockout markedly reduced the ability of SARS-CoV-2 to fuse with knockout host cells, thereby significantly impairing the infectivity of SARS-CoV-2 [56]. Interestingly, it was also found that furin cleavage of S proteins contributes to intracellular viral replication. In furin knockout cells, fewer virions were produced in SARS-CoV-2 infected cells at 72 h post infection, thereby verifying the role of furin in viral replication [56]. Additionally, it is important to note that SARS-CoV-2 variants differ in how effectively the spike protein is cleaved by furin at the S1/S2 boundary to prepare for viral entry. The Delta hallmark P681R substitution, which adds an additional basic residue adjacent to the multibasic furin-recognition motif, enhances furin’s ability to process the spike, resulting in more pre-cleaved spike on virions. This stronger furin processing promotes efficient cell-surface entry via TMPRSS2, greater cell–cell fusion via syncytia formation, and demonstrates higher pathogenicity in hamsters, while also improving replication fitness in human airway epithelial cultures [57,58]. In contrast, the Alpha-associated P681H variant produces only a modest increase in furin cleavage relative to Wuhan-Hu-1 [57]. Although P681H can influence spike processing and innate-immune sensitivity, it does not enhance fusogenicity or pathogenicity to the same extent as P681R [59,60,61]. As a result, the role and impact of cellular furin differs depending on the SARS-CoV-2 variant.

The utility of furin in clinical practice may extend beyond its potential as a therapeutic target. Smitha and Thomas (2023) also reveal furin as a potential biomarker for viral infection, specifically in the context of SARS-CoV-2; the study found increased furin levels by ELISA in the saliva of COVID-19 recovered and diabetic patients compared to healthy individuals [62]. Consequently, assessing furin levels in infected patients can serve as a novel diagnostic and therapeutic parameter, serving as a tool to guide decisions on the use of furin inhibitors during active infection. Collectively, the presented evidence supports furin’s vital role in various steps of the SARS-CoV-2 infectious cycle, including host -cell entry and replication. Therefore, creating an antiviral that inhibits the role of furin in the infectious cycle of SARS-CoV-2 is a promising avenue to explore further.

## 5. Role of Furin in DENV Infection

**Figure 3:**
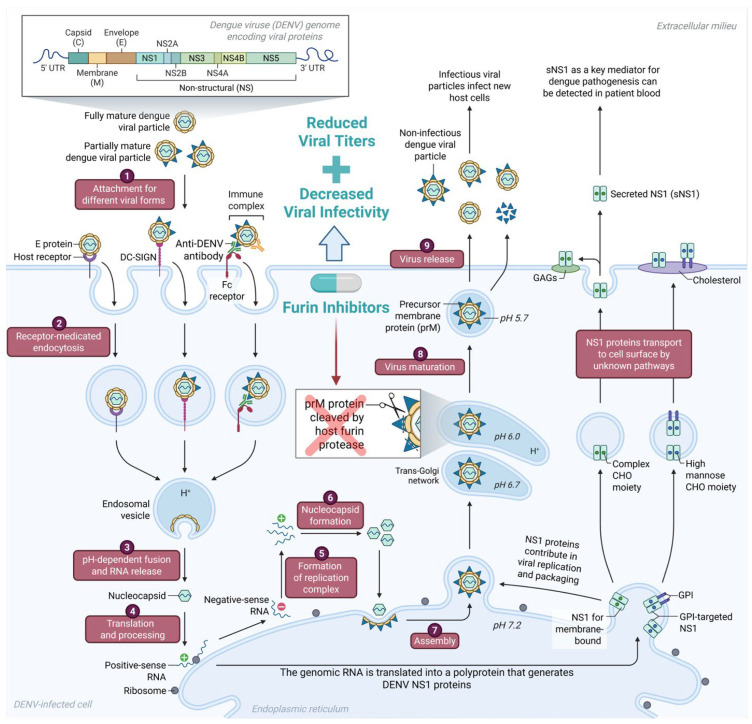
Role of furin protease in the DENV viral life cycle. Furin, localized in the trans-Golgi network (TGN), is essential for the maturation DENV particles through cleavage of the precursor membrane (prM) protein into a soluble “pr” fragment and a membrane-anchored (M) protein. This cleavage transforms immature, non-infectious particles into mature, infectious virions capable of efficient host cell entry. Inhibition of furin disrupts this process, leading to the release of immature or partially cleaved viral particles, thereby reducing DENV infectivity and propagation. Created in BioRender. Abdelhamid, A. (2026) https://BioRender.com/ekq21na (accessed on 27 February 2026).

Furin is proven to be an integral component of DENV’s replication cycle, making it a promising candidate in the management of DENV infection and co-infection with other viruses such as SARS-CoV-2. In DENV infected cells, furin is localized in the *trans*-Golgi network where it contributes to viral particle maturation prior to DENV release from infected host cells (Figure 3) [36]. Due to the change in pH environment from the endoplasmic reticulum to the mildly acidic *trans*-Golgi network, immature dengue virions undergo a structural change that exposes its premembrane protein (prM-E) for cleavage by the furin protease. This results in mature progeny with a membrane anchored (M) protein and a soluble “pr” fragment that dissociates only after exocytosis into neutral pH to prevent premature fusion in the secretory pathway [3,63]. Alternatively, functional studies have shown that without furin, uncleaved prM blocks the low-pH-triggered rearrangements of E glycoprotein required for membrane fusion, so virions that fail to undergo furin processing are poorly infectious [63,64]. Consistent with this, furin-deficient LoVo cells release virions with high prM content that are ~10^4^ -fold less infectious than furin-processed counterparts [64,65]. Moreover, population and single-particle analyses show that furin activity increases the fraction of mature particles [65] more enforced furin expression in producer cells (Vero-furin) drives more complete prM cleavage, thus more mature progeny with higher specific infectivity, and reducing sensitivity to antibodies that preferentially recognize immature virions [36]. While fully immature particles are generally non-infectious, anti-prM antibodies can render them infectious via Fcγ-receptor-mediated uptake, however its pathway still depends on host furin activity after entry [66]. Together, these observations position furin as a key host cell factor that greatly impacts DENV maturation and entry by coordinating prM cleavage and pH-dependent pr dissociation, making it a promising strategy for HAD therapy for DENV infections and co-infections.

## 6. Targeting Furin as an Optimal Antiviral Target Within PC Family

Before developing highly selective furin inhibitors as a broad-spectrum antiviral therapeutic, it is also important to validate why furin is targeted rather than other proteases in the same family. Relative to the other PC members, furin is the first identified and most extensively characterized, making it an ideal candidate for antiviral treatment [38]. Furthermore, of the PC members, furin and PACE4 mRNA exhibit a widespread tissue distribution, but only furin is ubiquitously expressed [67]. Furthermore, because furin is located in various areas in the cell, such as in the TGN and endosomal compartments, cell surface, and in extracellular spaces, this may explain its easy accessibility for viral hijack relative to the other PCs [49]. Unlike other PC members, furin remains an excellent candidate as it has been reported to process most viral surface glycoproteins and therefore will not only protect against SARS-CoV-2 and DENV, but several other viruses such as Marburg virus, Ebola virus, Influenza A virus, Hepatitis B virus, and Zika virus [49]. Additionally, the increased pathogenicity of H5 and H7 influenza A virus strains is associated with the polybasic HA cleavage motif that is cleaved by ubiquitously expressed furin-like convertases, as opposed to tissue-restricted proteases such as trypsin-like airway or gastrointestinal proteases [39,50]. Therefore, furin is an attractive antiviral target due to its ubiquitous expression and ability to recognize several viral glycoproteins.

## 7. Therapeutic Targeting of Furin in SARS-CoV-2

Several studies have evaluated furin inhibitors as potential antiviral agents for SARS-CoV-2 as shown in Table 1**.** Indeed, extensive preclinical work has shown that furin inhibition can effectively suppress SARS-CoV-2 replication, spike cleavage, and syncytium formation. Early studies identified *Aframomum melegueta* extracts containing bioactive compounds capable of inhibiting furin and suppressing SARS-CoV-2 replication *in vitro*, highlighting potential ethnobotanical resources for antiviral discovery [68]. Among synthetic inhibitors, MI-1851 has emerged as one of the most extensively studied molecules. It strongly inhibits viral replication in human airway epithelial cells and shows synergistic effects when combined with TMPRSS2 inhibitors, which target a complementary protease involved in spike activation [69]. Subsequent pharmacotoxicological evaluations confirmed MI-1851’s favorable safety profile and its potential as a lead compound for therapeutic use [70].

Another major class of furin inhibitors includes Decanoyl-RVKR-chloromethylketone (Dec-RVKR-CMK or CMK) and its related derivatives. CMK effectively blocks SARS-CoV-2 spike cleavage, reducing viral production and preventing the formation of pathogenic syncytia in infected cells [71]. Further studies confirmed CMK’s capacity to inhibit furin-mediated cleavage across multiple variants, including alpha, beta, gamma, delta, kappa, and omicron, thereby underscoring its consistent antiviral activity [72]. Interestingly, CMK was also shown to decrease viral activity even in spike mutants with altered furin cleavage sites (such as R682A), suggesting that its effects may extend beyond direct furin inhibition [73]. Similarly, naphthofluorescein was identified as another potent inhibitor that blocks spike protein processing and suppresses viral production [71]. Computational analyses demonstrated that naphthofluorescein exhibits greater binding stability and affinity for furin compared to CMK [74]. These findings, reinforced by additional *in silico* validation [75], position both CMK and naphthofluorescein as key furin inhibitors for potential drug development. Further research has introduced several new furin-targeting compounds. The BOS series inhibitors (BOS-318, BOS-857, BOS-981) were shown *in vivo* to reduce viral entry into lung cells when administered with a TMPRSS2 inhibitor, leading to an approximately 95% reduction in viral infection [76]. Notably, diminazene was identified as a dual inhibitor of furin and TMPRSS2 with IC_50_ values of 13.2 μM and 1.35 μM, respectively, offering a single-agent approach for blocking multiple host proteases involved in viral entry [77].

Natural and engineered molecules have also been explored as furin inhibitors. Computational efforts revealed that vitamin B12 exhibits strong furin-binding potential through docking and molecular dynamics simulations [78]. Theta-defensins, cyclic antimicrobial peptides, also showed favorable docking interactions with furin and were proposed as stable peptide-based inhibitors [79]. α-SNAP (alpha-soluble NSF attachment protein), an interferon-upregulated host protein, was experimentally shown to inhibit furin by binding to its P domain, thereby preventing the cleavage of SARS-CoV-2 spike and other furin-dependent glycoproteins [80]. Likewise, an engineered SerpinB3 variant possessing an anti-furin reactive center loop displayed concentration-dependent inhibition of SARS-CoV-2 pseudoparticle entry [81].

Several studies have also identified combinations and new molecular scaffolds with enhanced inhibitory activity. Kukoamine A, Zeaxanthin, and Clexane were recognized through *in silico* and *in vitro* screening as new furin inhibitors that potentiate the activity of CMK, improving its overall inhibitory efficiency [82]. Fucoidans, sulfated polysaccharides of marine origin, were shown to reduce SARS-CoV-2 viral loads in infected hamsters by inhibiting furin activity and blocking spike–cell interactions [83]. Interestingly, permethrin was found to act as a selective, non-competitive furin inhibitor that binds a novel allosteric pocket, suggesting a new class of protease-selective inhibitors [84]. High-throughput screening further identified a furin-targeting molecule that acts on secondary furin binding sites (exosites) rather than the catalytic pocket, offering potential improvements in selectivity and safety [85]. Nonetheless, a non-peptidic small molecule furin inhibitor, demonstrated comparable activity to established inhibitors (IC_50_ = 17.58 μM) and represents a promising drug-like scaffold for further drug optimization [86].

Several compounds were validated through *in silico* models that confirmed stable binding and favorable pharmacokinetic properties. In particular, luteolin, CMK, and naphthofluorescein exhibited stable interactions with the furin active site and favorable drug-likeness properties [75]. Collectively, these findings confirm that multiple furin-targeting strategies such as direct catalytic inhibition and allosteric modulation can effectively suppress SARS-CoV-2 infection *in vitro* and *in vivo*.

**Table 1:** Preclinical Studies Investigating Furin Inhibitors for SARS-CoV-2.

Author (Year) [Ref]	Furin Inhibitors	Study Type	Findings
Omotuyi et al. (2020) [68]	*Aframomum melegueta*	*In vitro*	Contains compounds that inhibit SARS-CoV-2, partially through furin inhibition.
Bestle et al. (2020) [69]	MI-1851	*In vitro*	Strongly inhibits SARS-CoV-2 replication in human airway cells; synergizes with TMPRSS2 inhibitors.
Cheng et al. (2020) [71]	Decanoyl-RVKR-chloromethylketone (Dec-RVKR-CMK) and naphthofluorescein	*In vitro*	Block spike protein cleavage, suppressing virus production and syncytium formation.
Cheng et al. (2021) [73]	CMK	*In vitro*	Further decreases in luciferase reporter activity of the R628A mutant, suggesting additional furin-independent effects warranting further investigation.
Negahdaripour et al. (2022) [79]	Theta-defensins	*In silico*	Docking analysis suggests that theta-defensins can function as furin inhibitors.
Zhang et al. (2022) [87]	Dec-RVKR-CMK	*In vitro*	Prevents infection by WT and ΔF mutant SARS-CoV-2; identifies K814A as novel furin functional site.
Singh et al. (2022) [81]	SerpinB3 (reactive centre loop variant)	*In vitro*	Exhibits concentration-dependent inhibition of SARS-CoV-2 pseudoparticle entry.
Wang et al. (2022) [80]	Alpha-Soluble NSF Attachment Protein (α-SNAP)	*In vitro*	Inhibits furin by interacting with its P domain; blocks spike protein cleavage.
Zaragoza-Huesca et al. (2022) [82]	Kukoamine A, Zeaxanthin, and Clexane	*In silico and in vitro*	Identified as new furin inhibitors; enhance CMK efficiency in blocking S protein proteolysis.
Essalmani et al. (2022) [76]	BOS-981, BOS-318, and BOS-857	*In vivo*	Combining with TMPRSS2 inhibitor achieves ~95% reduction of viral infection in lung cells.
Pandya et al. (2022) [78]	Vitamin B12	*In silico*	Docking and molecular dynamics simulations demonstrate strong inhibitory effects on furin.
Pászti-Gere et al. (2022) [70]	MI-1851	*In vitro*	Shows suitable pharmaco-toxicological parameters as potential COVID-19 drug candidate.
Xu et al. (2022) [77]	Diminazene	*In vitro*	Dual inhibitor of TMPRSS2 and furin (IC_50_: 1.35 and 13.2 μM, respectively).
Paul et al. (2022) [88]	Analog I (trypsin inhibitor), analog II (chymotrypsin inhibitor), and analog III (mirauclin-like protein)	*In silico*	Analog II most recommended among three plant protein analogs to disrupt furin–spike complex formation.
Reuter et al. (2023) [72]	CMK	*In vitro*	Inhibits ACE2-independent cell–cell fusions across alpha, beta, gamma, kappa, delta, and omicron variants.
Yang et al. (2023) [83]	Fucoidans	*In vivo and in vitro*	Decreases SARS-CoV-2 viral loads in infected hamsters; inhibits furin activity and viral entry in Calu-3 or Vero E6 cells.
Feng et al. (2023) [84]	Permethrin	*In silico and in vitro*	Binds novel furin allosteric pocket; non-competitive inhibition with good selectivity and specificity.
Jorkesh et al. (2024) [85]	3-((5-((5-bromothiophen-2-yl)methylene)-4-oxo-4,5 dihydrothiazol-2-yl)(3-chloro-4-methylphenyl)amino) propanoic acid (Compound P3)	*In vitro high-throughput screening*	Novel antiviral scaffold possibly targeting furin exosites rather than its catalytic pocket.
Jaganathan et al. (2024) [74]	Naphthofluorescein and Dec-RVKR-CMK	*In silico*	Naphthofluorescein shows greater stability and binding affinity as a furin inhibitor than CMK.
Kolarič et al. (2025) [86]	N-[4-(1,3-thiazol-2-ylaminosulfonyl)phenyl]-3-{(E)-5-[(2-methoxyphenyl)methylene]-4-oxo-2-thioxo-1,3-thiazolidin-3-yl}propionamide (Compound 4)	*In silico and in vitro*	Novel non-peptidic small molecule furin inhibitor with IC_50_ of 17.58 μM; represents starting point for non-peptidic antiviral drug design.
Saih et al. (2025) [75]	CMK, luteolin, and naphthofluorescein	*In silico*	All three inhibitors showed stable binding to furin in molecular docking studies and exhibited favorable drug-likeness properties.

Abbreviations: SARS-CoV-2, Severe acute respiratory syndrome coronavirus 2; COVID-19, Coronavirus disease 2019; TMPRSS2, Transmembrane serine protease 2; Dec-RVKR-CMK, Decanoyl-Arg-Val-Lys-Arg-chlororomethylketone; α-SNAP, Alpha-soluble N-ethylmaleimide-sensitive factor attachment protein; BOS, Boston pharmaceutical; IC_50_, Half-maximal inhibitory concentration; ACE2, angiotensin-converting enzyme 2.

## 8. Therapeutic Targeting of Furin in DENV

DENV relies on furin to cleave its precursor membrane (prM) protein during virion assembly, a step essential for producing mature, infectious particles [89]. Inhibiting this cleavage disrupts viral maturation and significantly reduces viral infectivity. Several compounds originally designed or tested for SARS-CoV-2 have parallel relevance for DENV due to their shared host dependency. A summary of the preclinical studies investigating furin inhibitors for DENV is shown in Table 2**.**

Luteolin has been characterized as a furin inhibitor with activity against DENV. It inhibits furin enzymatic activity in an uncompetitive manner (Ki = 58.6 μM), suppresses antibody-dependent enhancement (ADE)-mediated infection in human peripheral blood mononuclear cells (PBMCs), and has shown antiviral efficacy against all four DENV serotypes *in vitro* [90]. However, this activity is cell-line dependent: luteolin had strong antiviral properties against DENV in huh-7 cells, but less pronounced efficacy in HEK-293T, A549, and BHK-21 cell lines and no effect in Vero cells, suggesting that the antiviral mechanism may involve host cell-specific factors beyond direct furin inhibition [90]. Moreover, *in vivo* studies in mice revealed only moderate therapeutic effects. Daily administration of luteolin at a dose of 4 × 100 mg/kg led to a modest 2-fold reduction in viremia at day 3 post-infection. Notably, even at this high dosage, luteolin did not protect mice from lethal DENV infection [90]. This lack of efficacy may be attributed to low potency, poor bioavailability, or rapid metabolism, which are common limitations associated with flavonoid compounds like luteolin. Importantly, no additional virological studies on luteolin against DENV have been undertaken since 2017, highlighting a critical gap in understanding its therapeutic potential. Nonetheless, these findings indicate that while luteolin demonstrates proof-of-concept as a furin inhibitor with anti-DENV activity, its therapeutic efficacy *in vivo* remains limited and requires further investigation. Interestingly, luteolin has exhibited strong docking affinity to SARS-CoV-2 furin *in silico* [75], making it one of the few natural compounds with preliminary dual antiviral potential against both pathogens.

Synthetic furin inhibitors have also been explored in DENV models. MI-1148 demonstrated strong inhibition of viral replication in cell culture, reducing virus titers by over 1000-fold [91]. However, MI-1148’s early tetrabasic forms displayed a narrow therapeutic window in mice due to systemic toxicity. Later analogues with modified residue composition (replacing the P2 arginine residue with lysine) retained antiviral potency while lowering toxicity by twofold *in vivo*. These structure–activity refinements highlight how therapeutic safety can be improved without compromising antiviral efficacy.

## 9. Furin Inhibition as a with Luteolin Strategy for Co-Infection

Co-infection with SARS-CoV-2 and DENV has become a growing concern in tropical and subtropical regions, where both viruses often circulate simultaneously [93]. Because each virus depends on furin-mediated cleavage for its life cycle (SARS-CoV-2 for spike activation and DENV for prM maturation), therapeutically targeting furin presents a unified host-based strategy for managing dual infection [90]. However, co-infection with SARS-CoV-2 and DENV remains complex as the two viruses differ in tissue tropism and immune response. In DENV, immune processes such as ADE and dysregulated inflammatory responses may influence disease severity, whereas SARS-CoV-2, the contribution of furin to infectivity varies across variants and interacts with other entry pathways such as TMPRSS2 [57,58,90]. The therapeutic effect of furin inhibitors in co-infection may depend on infection stage, tissue involved, and varying host immune response. At the same time, this HDA strategy has important limitations due to furin’s role in host cell processes and ubiquitous nature; systemic furin inhibition may thus disrupt prohormone processing, cytokine maturation, coagulation-related pathways, and metabolic signaling [40,42]. Therefore, to minimize host toxicity, future furin-targeted therapies will require carefully dosed or selective tissue-targeted approaches.

**Figure 4:**
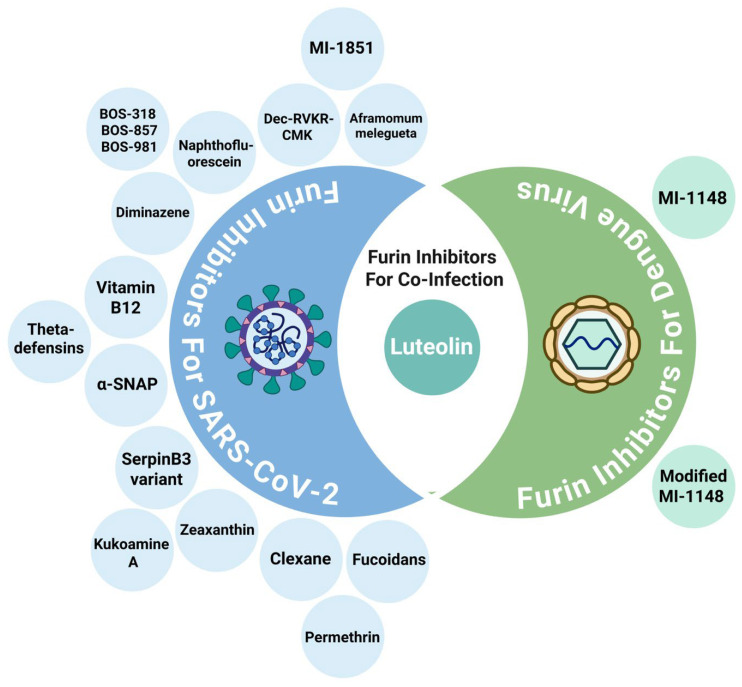
Visual representation of all furin inhibitors identified for SARS-CoV-2 and DENV infections. Although numerous compounds have demonstrated activity against either SARS-CoV-2 or DENV, Luteolin currently serves as a leading therapeutic for cross-pathogen targeting, with supporting experimental evidence of its antiviral activity against both SARS-CoV-2 and DENV. Created in BioRender. Abdelhamid, A. (2026) https://BioRender.com/vt6wn7e (accessed on 27 February 2026).

Despite these complexities, furin inhibitors remain promising for co-infection cases as it can impair SARS-CoV-2 spike activation and DENV prM maturation concurrently. To date, luteolin is the only compound with direct experimental evidence supporting antiviral activity against *both* SARS-CoV-2 and dengue virus (Figure 4). Luteolin is a naturally occurring flavonoid, chemically identified as 3′,4′,5,7-tetrahydroxyflavone. Structurally, it comprises a C6-C3-C6 framework with two aromatic benzene rings (A and B) linked by an oxygenated pyran ring (C), and features four hydroxyl (-OH) groups at carbon positions 5, 7, 3′, and 4′ [94,95]. It is an active metabolite isolated from numerous medicinal plants, including *Lonicera japonica*, and is abundant in various vegetables such as fresh spinach (*Spinacia oleracea*), parsley (*Petroselinum crispum*), and cabbage (*Brassica oleracea*), as well as in a range of fruits [96,97]. Plants rich in luteolin have been used in traditional medicine for centuries to treat various conditions [98,99]. Importantly, what makes luteolin particularly attractive for SARS-CoV-2 and DENV co-infection is that it possesses multi-faceted antiviral properties that extend beyond furin inhibition, and it has demonstrated activity against several animal viruses [100]. For instance, luteolin has been shown to inhibit the SARS-CoV-2 3-chymotrypsin-like protease (3CLpro), bind to the spike-S1 glycoprotein to block viral entry, and inhibit RNA-dependent RNA polymerase (RdRp) activity, which further support its therapeutic potential against SARS-CoV-2 [101,102,103]. In addition to its direct antiviral effects, luteolin exhibits potent anti-inflammatory and antioxidant activities, which are crucial for mitigating the cytokine storm and oxidative damage associated with severe viral infections [100]. Luteolin achieves this by modulating key signaling pathways such as JAK1/STAT3 and MAPK, and by inhibiting the NLRP3 inflammasome [104,105]. Its antioxidant capacity stems from its chemical structure, which enables it to donate hydrogen atoms from its hydroxyl groups to scavenge reactive oxygen species (ROS) and inhibit pro-oxidant enzymes like lipoxygenases and cyclooxygenases [95,106,107].

Despite its therapeutic promise, the clinical application of luteolin is hampered by its poor aqueous solubility, limited permeability, and suboptimal oral bioavailability, which may explain luteolin’s weak effect *in vivo* in the context of DENV infection [90,108,109]. In nature, luteolin is predominantly found in glycosylated forms (e.g., Lut-7-O-glucoside) [95]. Following oral administration, these glycosides are hydrolyzed to yield the free aglycone form of luteolin, which is then absorbed [110]. Pharmacokinetic studies have shown that while gastrointestinal absorption is relatively efficient, with peak plasma concentrations achieved within 1–2 h post-administration, luteolin undergoes extensive first-pass metabolism in the intestinal and hepatic cells [111,112]. The reported bioavailability of total luteolin is 53.9%; however, the actual bioavailability of the free, active form is merely 17.5%, as it is rapidly converted into conjugated metabolites such as luteolin glucuronides, sulfates, and methylated derivatives (e.g., chrysoeriol and diosmetin) [95,113,114]. Following absorption, luteolin and its metabolites are rapidly and widely distributed throughout the body, with high concentrations found in the liver, kidneys, gastrointestinal tract, and lungs [95]. Elimination occurs primarily through biliary excretion, with metabolites such as luteolin-3′-O-glucuronide being excreted in urine. A secondary peak in plasma concentration observed 2–8 h post-administration suggests enterohepatic recirculation [95,115,116].

To be used as an antiviral, potential routes of administration for luteolin could include oral, parenteral, or inhalational delivery. The optimal route of luteolin administration will likely depend on the target infection. For respiratory SARS-CoV-2 infection, inhalation delivery may offer the advantage of achieving high drug concentrations directly in airway epithelia. Conversely, for dengue, oral administration would be the most patient-friendly option but faces significant bioavailability challenges, potentially necessitating parenteral routes (e.g., intravenous) in acute severe dengue to ensure therapeutic plasma concentrations. However, each of these suggested approaches requires dedicated pharmacological studies to confirm therapeutic efficacy and optimize dosing.

Promisingly, the pharmacokinetic limitations of luteolin can be addressed through the use of modified chemical forms of luteolin innovative drug delivery systems and combination therapies. Miao et al. (2021) [117] formulated luteolin-loaded methoxy poly(ethylene glycol)-polylactide micelles (luteolin/MPEG-PLA) to enhance bioavailability in pulmonary infectious diseases, while Ren et al. (2023) [118] achieved a four-fold increase in luteolin bioavailability using a cyclodextrin metal–organic framework-modified dry powder inhaler for treating fibrotic interstitial lung disease. In addition, various pharmaceutical techniques have been employed to enhance its solubility and bioavailability, including cyclodextrin complexation, polymeric micelles, phospholipid complexation, and nanoencapsulation [119,120,121,122]. For example, inhalable hyaluronidase nanoparticles (Lut@HAase) have been designed for targeted lung delivery, improving penetration into fibrotic lesions [123]. Similarly, luteolin-loaded long-circulating micelles (DTLLM) and luteolin-loaded nanovesicles (LT-NV) have demonstrated sustained drug release and significantly improved dissolution rates compared to free luteolin [124,125]. Hence, similar strategies could be utilized to improve luteolin’s pharmacokinetics and support its therapeutic potential in the context of SARS-CoV-2 and DENV. Furthermore, combining luteolin with other bioactive compounds or trace elements offers another avenue to enhance its therapeutic effect. In the context of COVID-19, combination with zinc and magnesium synergistically inhibits SARS-CoV-2 3CLpro expression and helps modulate the cytokine storm [103]. Co-occurring metabolites such as eriodictyol have also been shown to enhance the intestinal absorption of luteolin, further improving its bioavailability [95,126]. Collectively, these combination strategies can increase the bioavailability of luteolin and therefore improve its overall therapeutic effectiveness.

Regarding safety, pure luteolin is generally considered devoid of significant toxicity for human consumption, consistent with its long history of use in traditional medicine and its presence in the human diet. However, a comprehensive evaluation is necessary, as some studies indicate that high doses may lead to adverse effects. *In vitro* evaluations have demonstrated some elements of toxicity, with concentrations of 100 μM affecting cell growth in human retinal microvascular endothelial cells and 2.5 μM reducing viability in human lymphoblastoid TK6 cells [127,128,129]. Luteolin exposure has also been linked to glutathione depletion and the formation of reactive ortho-quinone metabolites, which have been associated with cytotoxicity in primary rat hepatocytes [130]. Furthermore, because luteolin and its metabolites can cross the placental barrier, caution may be warranted regarding fetal exposure during pregnancy [131]. While the available literature suggests that luteolin is a comparatively safe natural compound, the lack of comprehensive *in vivo* toxicity evaluations presents a research gap. Therefore, rigorous *in vivo* toxicity studies are essential to establish a definitive safety profile and guide the secure development of luteolin-based pharmaceuticals for clinical applications.

Taken together, while luteolin currently stands out as the only furin inhibitor with demonstrated dual activity against SARS-CoV-2 and DENV, its path to clinical use requires a multi-faceted approach. Given the significant research gap, further studies investigating luteolin, particularly in the context of DENV, are warranted to fully elucidate its therapeutic potential. Moreover, studies should explore whether combining luteolin with other host protease inhibitors such as TMPRSS2 blockers can enhance antiviral efficacy while minimizing required doses. Additionally, future research should prioritize investigating the development of optimized nanoformulations and combination therapies to overcome luteolin’s bioavailability challenges, alongside rigorous pharmacological and toxicological *in vivo* studies. These efforts will be crucial to fully harness the therapeutic potential of this promising natural compound and translate this dual-inhibition concept into a clinically viable treatment. As current evidence stands, luteolin remains the only validated compound demonstrating furin-targeted inhibition against both SARS-CoV-2 and DENV, positioning it as a promising lead compound for managing their co-infection.

## 10. Safety Considerations and Toxicity of Furin Inhibition

Furin, as previously discussed, is a ubiquitously expressed PC that plays a critical role in a multitude of physiologic processes, including cellular signaling and immune regulation, which are essential for the maintenance of systemic homeostasis. Given this broad and multi-faceted involvement, it is reasonable to anticipate that furin inhibition may be associated with a potentially unfavorable safety profile that may negate clinical applicability despite its promising antiviral potential.

Indeed, a primary concern relates to the furin inhibition-mediated immune dysregulation. This concern is driven by furin’s crucial role in immune regulation, in which, it has been shown to serve as a pro-TGFB-1 (transforming growth factor beta) converting enzyme [31]. Through this, furin becomes an important mediator of regulatory T cells (Treg), which play an essential role in immune regulation, and thus prevention of autoimmune disease [31]. Substantiating this role in immune regulatory processes, furin inhibition has been shown to disrupt peripheral tolerance and induce autoimmune reactions in mice [31]. These pro-autoimmune effects of furin inhibition have also been observed in human-derived cells [132]. Indeed, in fibroblast-like synoviocytes derived from patients with rheumatoid arthritis, it has been shown that furin inhibition resulted in increased invasiveness and expression of interleukin-1β and tumor necrosis factor-α [132]. These cytokines are key drivers of rheumatoid arthritis pathogenesis and are implicated in the progression of multiple autoimmune diseases [133,134]. Collectively, this experimental evidence reveals that although furin represents a promising target a broad-spectrum antiviral strategy, its inhibition may disrupt key immune regulatory pathways. This raises the possibility of precipitating autoimmune responses in individuals with underlying quiescent disease or exacerbating pathology in those with established autoimmune conditions.

Furin inhibition may also interfere with normal cellular growth, tissue remodeling, and extracellular matrix regulation. Indeed, furin is required for the activation of multiple substrates, including matrix metalloproteinases and growth factors [31,43]. Disruption of these pathways may lead to altered cell migration, impaired tissue repair, and dysregulated angiogenesis [135]. For example, inhibition of furin activity has been shown to affect the maturation of proteins such as vascular endothelial growth factors and metalloproteinases, which are critical for cell signaling and tissue remodeling processes [136]. While such effects may be therapeutically beneficial in cancer settings, they may also result in unintended consequences in normal physiological contexts.

Furthermore, the ubiquitous expression and broad substrate specificity of furin raise concerns regarding multi-organ and systemic effects. Because furin participates in the activation of numerous proteins across endocrine, cardiovascular, and metabolic pathways, its inhibition may lead to widespread disruptions in physiological homeostasis [40,42]. These pleiotropic effects are difficult to predict and may vary depending on tissue type, disease state, and the specificity of the inhibitor used.

Overall, despite the lack of comprehensive studies addressing the safety profile of furin inhibitors, the central role of furin multiple physiologic pathways suggests that its inhibition may be associated with several safety concerns. Nevertheless, further experimental and clinical studies are imperative to systematically evaluate the safety of furin inhibition and to determine whether the aforementioned theoretical risks are translated *in vivo* and clinical settings.

## 11. Conclusions

Overall, furin inhibitors have emerged as promising broad-spectrum antiviral candidates through their inhibition of the host-derived protease furin, an enzyme exploited by multiple viral families throughout their infectious cycles. Our review highlights a practical lead, luteolin, as a key candidate with experimental activity, albeit preliminary, against both SARS-CoV-2 and DENV to date, relevant for managing co-circulation and co-infection settings. However, evidence supporting luteolin’s therapeutic efficacy against DENV *in vivo* remains weak potentially due to luteolin’s poor pharmacokinetic profile and hence requires further investigation of methods to overcome this limitation. More importantly, no studies to date have investigated the use of luteolin or other furin inhibitors in co-infection models, highlighting a crucial research gap in this context. This gap extends beyond SARS-CoV-2/DENV co-infection, as other clinically relevant scenarios such as COVID-19 and influenza co-infection, may also represent potential applications for furin-targeting therapies, given the shared reliance of multiple respiratory viruses on host protease activation pathways. This review therefore serves not as definitive validation of clinical utility but as a plea for complementary virological studies, including: (1) *in vivo* efficacy studies of furin inhibitors in appropriate animal models for SARS-CoV-2 and DENV, particularly co-infection; (2) pharmacokinetic profiling to address the bioavailability limitations that likely contributed to luteolin’s modest outcomes against DENV; (3) the investigation and validation of optimized nanoformulations and combination therapies designed to overcome luteolin’s bioavailability challenges; (4) the determination of optimal luteolin dosing and route of administration; (5) rigorous toxicological *in vivo* studies to confirm luteolin’s safety; and (6) the investigation of combining furin inhibitors with existing direct-acting antivirals to improve therapeutic efficacy, as current evidence on such approaches remains limited. Given that furin is a host-dependent enzyme with various physiological functions, systemic inhibition carries the risk of potential adverse effects. Consequently, future research should aim to develop selective furin inhibitors that specifically target virus-associated furin activity while minimizing interference with normal cellular processes, thereby improving safety and enhancing clinical applicability. Advancements in structural biology and computational modeling offer additional promises for optimizing furin inhibitor therapy by improving drug bioavailability, reducing systemic toxicity, and enabling targeted delivery. Continued investigation in this area is crucial to support their successful translation into clinical practice to better prepare health systems for concurrent and future outbreaks.

## Figures and Tables

**Table 2:** Preclinical studies investigating furin inhibitors for DENV.

Author (Year) [Ref]	Furin Inhibitor	Study Type	Findings
Peng et al. (2017) [90]	Luteolin	*In vitro* and *in vivo*	Luteolin is a furin inhibitor with antiviral activity across all four DENV serotypes *in vitro* and suppresses ADE-mediated DENV infection in human PBMCs. Luteolin inhibited the furin enzyme activity in an uncompetitive manner (Ki = 58.6 μM). Luteolin also exhibited *in vivo* antiviral activity resulting in moderately reduced viremia; however, it did not protect mice from lethal DENV infection.
Kouretova et al. (2017) [91]	MI-1148 (Compound 46)	*In vitro*	A strong inhibition of DENV replication in cell culture was observed for the specific furin inhibitors (especially MI-1148), which reduced virus titers more than 1000-fold.
Ivanova et al. (2017) [92]	Modified form of MI-1148 (lysine instead of P2 arginine residue)	*In vitro* and *in vivo*	MI-1148 (tetrabasic compound) has a narrow therapeutic range in mice. Significantly reduced toxicity was observed for some tribasic analogues. Replacing P2 arginine in MI-1148 with lysine led to slightly decreased potency, but similar antiviral activity against DENV in cell culture and twofold decreased toxicity in mice.

Abbreviations: DENV, Dengue virus; ADE, Antibody-dependent enhancement; Ki, Inhibition constant.

## Data Availability

Data sharing does not apply to this article, as no new data was created or analyzed.
